# Medico-legal considerations on “Lotus Birth” in the Italian legislative framework

**DOI:** 10.1186/s13052-019-0632-z

**Published:** 2019-03-18

**Authors:** Alessandro Bonsignore, Francesca Buffelli, Rosagemma Ciliberti, Francesco Ventura, Andrea Molinelli, Ezio Fulcheri

**Affiliations:** 1 Department of Health Sciences (DISSAL) – Section of Legal and Forensic Medicine and Bioethics, University of Genova, Via De Toni 12, 16132 Genoa, Italy; 2 Ph.D Course in Paediatric Science, Fetal-Perinatal and Paediatric Pathology, University of Genova, Genoa, Italy; 3 Fetal and Perinatal Pathology Unit, IRCCS Istituto Giannina Gaslini, Largo G. Gaslini, 16147 Genoa, Italy; 4 Department of Surgical Sciences and Integrated Diagnostics (DISC) – Pathology Division of Anatomic Pathology, University of Genova, Largo Rosanna Benzi 10, 16132 Genoa, Italy

**Keywords:** Lotus birth, Placenta, Medico-legal implications, Italian legislative framework, Health safety

## Abstract

The term “Lotus Birth” identifies the practice of not cutting the umbilical cord and of leaving the placenta attached to the newborn after its expulsion until it detaches spontaneously, which generally occurs 3–10 days after birth. The first reported cases of Lotus Birth date back to 2004 in Australia.

Supporters of such a procedure claim that the newborn is better perfused, endowed with a more robust immune system and “less stressed”.

However, it should be pointed out that histopathological study of the placenta is increasingly being requested in order to investigate problems of an infective nature or dysmaturity affecting the foetus, and situations of risk affecting the mother. Moreover, from the legal standpoint, there is no uniform position on the question of whether the placenta belongs to the mother or to the newborn. Lastly, a proper conservation of the embryonic adnexa is very difficult and includes problems of a hygiene/health, infectivological and medico-legal nature.

The authors analyzed all these aspect in the Italian legislative framework, reaching the conclusion that Lotus Birth is inadvisable from both the scientific and logical/rational points of view.

## Background

Cutting the umbilical cord is a medical act which – first by tradition and then on scientific bases – is routinely implemented in obstetric centres in all western countries. In recent years, however, a school of thought that is opposed to this tradition has emerged.

According to this opposing view, birth is seen from the standpoint of the foetus, and it is claimed that clamping the cord while it is still pulsating could be harmful to the newborn. In this regard, some authors [1] maintain that it would be more proper to delay closure of the vessels of the cord at least for a few minutes, so that much of the blood contained in these vessels (foetal blood) can flow back from the placenta; in this way, the newborn would be provided with a maximal reserve of iron and haemoglobin. This means waiting until the cord has spontaneously ceased to pulsate, rather than interrupting the foetal-placental circulation and, with it, the supply of oxygen that still reaches the newborn through the blood in the cord. In this case, the cord would be cut only when its natural functions have ceased. This argument has partly given rise to the theory of the so-called “Lotus Birth”.

The term was coined in 1979 to identify the practice of not cutting the umbilical cord and of leaving the placenta attached to the newborn after its expulsion until it detaches spontaneously, which generally occurs 3–10 days after birth [2]. According to the advocates of this method, the foetus and the placenta are formed from the same cells, and are therefore a single unit. Thus, if the newborn is not artificially separated from this part of itself, it will be endowed with a more robust immune system, as all the “vital force” contained in the placenta and a considerable amount of blood will be conveyed to it through the umbilical cord. Even babies delivered by means of caesarean section are said to benefit. Moreover, supporters of this method claim that, if the mother has suffered emotional trauma or stress during pregnancy, the baby will not display signs of “residual stress”; indeed, babies born in this way are described as “calm and well-balanced”: in short, “born with… the placenta”.

From the practical standpoint, this technique requires the mother to take her newborn baby home and to procure a sieve of appropriate size, which will be placed in a bowl and in which the placenta will be kept.

The placenta will be preserved in this way for a minimum of two days up to a maximum of two weeks, during which time it will be treated with sea salt and ginger in order to improve its conservation and, at the same time, reduce the unpleasant smell that a decomposing organ inevitably produces.

The first reported cases of Lotus Birth date back to 2004 in Australia [3]. In Italy, it is estimated that about 100 women per year request this so-called “integral birth” [4].

This paper aims at providing an analysis of clinical risks, bioethical issues and medico legal aspects concerning such a procedure, in the Italian legislative framework.

## The placenta

Embryo-foetal adnexa are defined as all those structures of the product of conception that are not part of the embryo or of the foetus. Among these, the most important in terms of its functional and morphological complexity is undoubtedly the placenta, which consists of the funiculus, the chorial disc and free membranes. The human placenta is a highly differentiated organ, which is essential for the oxygenation, hydration and nutrition of the foetus, in addition to carrying out a complex endocrine activity.

With regard to its structural aspects, the chorial disc comprises the cotyledons (made up of villous branches), the chorion and part of the amniotic sac (foetal side of the chorial disc). On the maternal side, at the apex of the cotyledons, there are more or less extensive flaps of basal decidua that remain attached at the moment of expulsion.

In the gravid uterus, the basal decidua is composed of a spongy layer that borders on the myometrium and a compact layer made up of decidualised stromal elements and a few cells of trophoblastic origin (extra-villous intermediate trophoblast). The basal decidua is separated from the cotyledons that make up the chorial disc by a fibrinoid layer, called Nitabuch’s stria [5]. The vascular structure of the chorial villi derives from, or merges into, the amniochorial vessels, and is constituted by the stem vessels and their finer branches, down to the capillaries of the exchange villi.

The placenta is therefore an organ of a “haemochorial” villous nature [6]. in which the foetal blood is separated from the maternal blood by a “barrier” made up of five tissues, all of foetal origin: the endothelium of the capillaries, the basal membrane of the endothelium, the stromal connective tissue of the villus, the basal membrane of the trophoblast, and the villous trophoblast (cyto- and syncytiotrophoblast) in direct contact with the maternal blood circulating in the cotyledon.

Situated above the chorial layer, the amnion lines the foetal side of the placenta and folds back on the funiculus, enveloping it up to its insertion at the umbilicus of the foetus, where it is in continuity with the skin. A thin layer of decidua modified by the extra-villous intermediate trophoblast remains, instead, adherent to the maternal side of the chorial disc [7].

As mentioned above, the placenta is composed of the amniotic sac, the funiculus and – predominantly – the chorial disc, and accompanies the foetus up to the time of birth. Only then does the newborn become separated from the placenta, thus abandoning that part of himself that was previously indispensable, and with which he constituted an inseparable unit – the foeto-placental unit [8].

First of all, it should be pointed out that histopathological study of the placenta is increasingly being requested in order to investigate problems of an infective nature or dysmaturity affecting the foetus, and situations of risk (mainly linked to hypertension, dysendocrine/dysmetabolic or dysreactive states) affecting the mother [9]. Indeed, the placenta is the organ of exchange between the mother and the foetus, and, in the presence of haemodynamic alterations, it can adapt, modify or markedly alter its structure, suffering damage of variable extent and degree.

This adaptability and the functional modulations of the chorial disc largely depend on the action of the extra-villous intermediate trophoblast, which develops in the basal decidua and modifies, among other things, the structure of the spiral arteries and of the blood lacunae.

The close intermixing between the extra-villous component of the trophoblast and the maternal decidual structures (especially stromal and vascular) has given rise to the idea that the basal decidua (maternal portion) is part of the placenta.

This long-standing notion is erroneous and is rooted not only in medical-scientific and obstetric thinking, but also, and even more so, in the popular perception, where it has even influenced the world of culture, art and literature.

This erroneous conviction is strengthened by the fact that, from a legal standpoint, under the provisions of art. 7 of Italian D.P.R. 285/90 (Mortuary Police Regulations currently in force, though now undergoing parliamentary reform), up to the 28th week of gestation the so-called “*products of conception*” (gestational age below 20 weeks) and the “*products of abortion*” (gestational age from 20 to 28 weeks) are considered to be “part of the mother” and not separate individuals with their own biological-human and personal dignity. Thus, the placenta is generally regarded as a maternal organ, from which the foetus detaches only when it is capable of autonomous life, i.e. when it acquires a potential juridical personality (de facto after the first spontaneous breath) and, therefore, the right to its own individuality.

This means that the newborn is not granted possession of an organ which belongs to him and which can actually influence his postnatal development, so much so that foetal pathologies related to severe placental alterations, regardless of the condition of the mother, have an impact on the development and growth of the newborn and, later, of the child [8]. Thus, the placenta must be regarded biologically as an autonomously defined organ. As yet, however, from the legal standpoint, there is no uniform position on the question of whether the placenta belongs to the mother or to the newborn.

This ambiguity means that the human placenta, once expelled, is not usually considered to be of pertinence to a human organism (whether mother or newborn, as in the case of a blood sample from the umbilical cord). It therefore becomes extraneous to the juridical property of the puerpera, and is entrusted to the healthcare facility for destruction or utilisation for diagnostic purposes.

From the juridical point of view, this issue has been addressed by several authors. One of these, Mantovani [10], claims that, once the foetal adnexa have detached from the body of the subject, they may, in general terms, be regarded as having ceased to belong to the sphere of the rights of the person and become a *res*.

This notion, however, immediately raises the question of the property right of the subject from whose body the *res* has detached, in that this very detachment constitutes a mode of origin of ownership whereby body parts are not rendered *res nullius*. Moreover, according to the author, it seems that this property right should be granted with regard to any part of the body, in that there is no reason to distinguish one body part from another on the basis of either its functional importance or the way in which it is separated from the body. Only the individual subject has the freedom to regard a body part as his or her own, and hence either to keep it and to exercise the right of property over it or else to abandon it. In the case of abandonment, detached parts of the human body become *res derelictae* and therefore *res nullius*; at this point, they will either be destroyed – if they are of no scientific or diagnostic interest – or accrue to the property right of the healthcare facility, if they are of diagnostic, scientific, therapeutic or pharmaceutical interest. Thus, for what concerns the embryonic adnexa, the following considerations can be made:

1. such anatomical parts, at the moment of detachment from the puerpera, become the property of the puerpera herself, perhaps also because she is the guardian of a minor over whom she exercises parental responsibility (together with the father);
2. the parents are entitled to receive the adnexa from the healthcare facility or to have them examined elsewhere at their own expense (e.g. in a private clinic);
3. if the mother and father abandon them, the adnexa become the property of the healthcare facility, of which they are at the complete disposal.

Should the parents request that the embryonic adnexa be consigned to them for some need of their own or of the newborn, the adnexa would become a specific good which, as such, cannot but constitute an object of their property right. Thus, the problem lies rather in the real possibility to exercise this right at the practical level; it follows that the achievement of this objective requires at least two presuppositions:

1. proper conservation of the embryonic adnexa;
2. the absence of contraindications to conservation itself, as the adnexa do not constitute a biological product falling within the provisions of article 184 of the unified body of Italian health legislation (Italian R.D. 1265/34).

## Medico-legal considerations

In the light of what has been said, there necessarily emerge problems of a hygiene/health, scientific, infectivological [11]. and medico-legal nature, in addition to issues concerning the management of a newborn to whom the umbilical cord and placenta remain attached for a variable period of time.

It should be pointed out that, from the standpoint of the law and hygiene/health, there is no reference legislation that may suggest any conceivable use of the placenta, other than consignment to the healthcare facility [12]. Likewise, jurisprudence contains no decisions that contemplate the concept of the juridical availability of the placenta, if not indirectly through generic references to embryonic adnexa, chiefly with regard to the embryo in its entirety and to stem cells [13–15].

From the medico-legal point of view, objections inevitably arise, in that, before consenting to this practice, it is necessary to create a specific model of information – one which can reconcile the needs of the mother (and father) with the juridical norms and the scientific, hygienic and deontological demands.

Today, with the progressive modification of relationships between the hospital facility and the patient – to the extent of creating a so-called “social contact” – two opposing sets of rights have emerged; on the one hand, patients have the right to expect not only that their health be safeguarded, but also that their will be respected; on the other hand, the healthcare facility is entitled to safeguard its own interests and its employees. In the light of this consideration, it would be necessary to draw up a document, to be signed by the mother and father before delivery of the baby, in which they express the wish to follow the Lotus Birth protocol, on condition that the healthcare facility decides to endorse the request and is able to ensure proper execution of the technique.

Nevertheless, it must be borne in mind that – once the possibility to adopt this protocol has been agreed upon – if the need arises to carry out a diagnostic investigation regarding any condition whatsoever of the newborn or of the mother (previous or emerging pathology), the healthcare facility would be totally unable to perform any histopathological diagnostic examination of the placenta, notwithstanding the fact that any such examination would be right and proper and implicit in the above-mentioned contractual relationship between the hospital and the patient. Thus, it clearly emerges that, in such situations, another document would need to be signed, again before the birth of the baby, in which the parents express their wish to forgo any possible diagnostic examination, even if there is a concrete risk to the health of the newborn or the mother.

In this regard, the need has been felt in recent years in Italy to create new procedures for monitoring the quality of the healthcare services provided and the risks connected with them. In the field of neonatology, for instance, the Ospedale Policlinico San Martino-IST in Genova, Italy, as part of its routine evaluation of clinical risk, has adopted the practice of adequately conserving the placenta until the newborn is discharged; in this way, it is possible to carry out any histological investigation that may be deemed necessary in the event of the emergence of any unforeseeable neonatal symptoms in the immediate *post-partum* period.

If the principles dictated by the Lotus Birth protocol were to be applied, it would no longer be possible to carry out such investigations, owing to the inadequate conservation of the placenta. Thus, in order to avoid any conflict between the hospital’s policy of clinical risk management and the will of the baby’s mother and father, a third document would need to be signed by the couple; this would exonerate the hospital from responsibility for any untoward consequences that might arise – especially in terms of damages – should any failure to diagnose any neonatal pathology be attributable to the non-examination of the placenta.

Finally, in addition to the issues of responsibility evoked by implementation of the Lotus Birth protocol, it must also be noted that the scientific bases on which the practice is founded are scant, indeed almost non-existent.

## Ethical considerations

The issue of performing the Lotus Birth, on maternal request, also solicits ethical considerations.

The motivation towards choosing the Lotus Birth is the achievement of a natural birth. Cutting the umbilical cord is, indeed, seen as a violent act.

Moreover, a woman may request the Lotus Birth practice in order to exercise her right to individual choice and self-governance. However, the ethical principle of autonomy also requires that the woman (or both parents, if the woman authorize it) is/are adequately informed and aware of the implications underlying such a choice.

First of all, the physicians will inform the mother about the fact that the Lotus Birth requires the primary caregiver remaining close to a bag of decomposing flesh, and it keeps her homebound as she cares for the newborn until the umbilical cord detaches.

In addition, physicians should caution that in the literature there is no compelling evidence that the baby benefits from having a discarded organ attached to him/her for days, while, there is a lack of research regarding its safety [16].

As a matter of fact the newborn is at a very delicate stage and he/she is very prone to diseases as his/her immune system is not developed so far. By keeping the umbilical cord attached, the environmental micro-organisms have a big chance of affecting the dead tissue.

Accordingly, the control over one’s own body and individuality, expressed in the increasing appreciation of the ethical principle of autonomy, cannot separate itself from the careful evaluation of the principles of non-maleficence and beneficence [17]. These principles involve not only the duty not to harm, but also the obligation to protect vulnerable groups and people.

Furthermore, the prudent assessment of benefits, burdens, and harms in health care decision-making impose the exclusion of the application of this procedure in the hypothesis in which the child is at high risk [18].

In fact, if it is true that certainty is not always valid in the medical field, in the hypothesis of certainty of risks to the health of the child, this procedure appears ethically inadmissible.

In this sense a thorough, rigorous and responsible information to the community can help to promote a culture based on scientific evidence, removing uncritical beliefs.

## Conclusions

In conclusion, in the light of what has been expounded above, it can be claimed that the practice of Lotus Birth is inadvisable from both the scientific and logical/rational points of view. If, however, we wish to underline the only meritorious principle of this procedure, i.e. the emphasis placed by its advocates on the importance of safeguarding the blood supply of the newborn, we may mention the following aspects:

1. given that the placenta is a reservoir of blood for the newborn, at the moment of delivery and in the early *post-partum* period, care must be taken to manoeuvre the baby correctly in relation to the position of the placenta, which, immediately after delivery, is still an integral part of the newborn’s blood circulation. Indeed, brusquely lifting the baby up (for the mother to see) will cause a rush of blood towards the placenta, which, at that moment, is in a lower position. This may result in rapid anaemia of the newborn. Indeed, it is for this very reason that great care has always been taken to clamp the funiculus while maintaining the placenta on a higher plane than the newborn, so that the baby will not be deprived of foetal blood;
2. that said, inappropriate mobilisation of the newborn, or of the placenta, without clamping, could cause deficient neonatal perfusion.

On the other hand, it must also be pointed out that, if the Lotus Birth “guidelines” are followed to the letter, the lack of clamping could give rise to a potential thrombotic risk, in that the establishment of a low-flow, low-resistance circulation, like that of the foetus-placenta *post-partum* could facilitate the formation of clots. Similarly, cases of idiopathic neonatal hepatitis following Lotus Birth have been described in the Literature [19]. These aspects require further investigations and studies, accordingly to the following opinion of the Royal College of Obstetricians and Gynecologists, expressed in 2008: “*at present, the practicee of lotus birth is new to the UK and there is a lack of research regarding its safety*” [16].

Nevertheless, regardless of the above-mentioned risks, it would seem that the healthcare facility cannot refuse to consign the placenta to the mother if she explicitly requests it, unless doing so were to engender a risk for the hygiene of the hospital environment and for public health (according to Italian D.P.R. 254/03: “*Regulation governing the management of medical waste pursuant to Article 24 of the Law of 31 July 2002, n. 179*”). In this regard, it should be borne in mind that non-compliance with hygiene/health provisions is a penally prosecutable offence under the terms of article 650 of the Italian Penal Code regarding provisions of public authorities. This may constitute a compelling disincentive for expectant mothers to pursue the logic of Lotus Birth.

Lastly, the importance of adopting informed forms is crucial, and a mutidisciplinary group is working on it.

## Abbreviations

D.P.R: Decree of the President of the Republic
RD: Royal Decree
